# Preparation and Study of Physicochemical and Adsorption Properties of Alginate Composites

**DOI:** 10.3390/ma18030629

**Published:** 2025-01-30

**Authors:** Małgorzata Wasilewska, Sylwia Gubernat, Paulina Gil-Kulik

**Affiliations:** 1 Department of Physical Chemistry, Institute of Chemical Sciences, Maria Curie-Sklodowska University, Maria Curie-Sklodowska Sq. 3, 20-031 Lublin, Poland; 2 Inżynieria Rzeszów S.A., ul. Podkarpacka 59A, 35-082 Rzeszów, Poland; sylwiagubernat44@gmail.com; 3 Department of Clinical Genetics, Medical University of Lublin, 11 Radziwillowska Str., 20-080 Lublin, Poland; pgil.poczt@vp.pl

**Keywords:** alginate–carbon/cellulose biocomposite, physicochemical characteristics, adsorption of drugs

## Abstract

This paper presents the preparation and study of the properties of alginate materials, which were obtained on the basis of sodium alginate, activated carbon, cellulose, and calcium chloride. Alginate–carbon (AlgCa + C) and alginate–cellulose (AlgCa + Cel) composites, as well as pure calcium alginate (AlgCa) for comparative purposes, were obtained. Their textural (nitrogen adsorption/desorption isotherms), morphological (scanning electron microscopy), thermal (thermal analysis), and acid–base (pH drift method) properties, as well as the swelling index, were investigated. Additionally, to determine the adsorption properties, comprehensive equilibrium and kinetic studies of the adsorption of sodium salts of ibuprofen (IBP), diclofenac (D), and naproxen (NPX) from aqueous solutions on biocomposities were carried out. Adsorption isotherms were fitted using the Marczewski–Jaroniec isotherm equation (R^2^ = 0.941–0.988). Data on the adsorption rate were analyzed using simple kinetic equations, of which the best quality of fit was obtained using the multi-exponential equation (R^2^ − 1 = (3.9 × 10^−4^)–(6.9 × 10^−4^)). The highest obtained adsorption values were reached in systems with alginate–carbon composite and were 1.23 mmol/g for NPX, 0.81 mmol/g for D, and 0.43 mmol/g for IBP. The AlgCa + C material was characterized by a large specific surface area (1151 m^2^/g), a high degree of swelling (300%) and high resistance to high temperatures.

## 1. Introduction

Water pollution constitutes a significant environmental and public health issue, primarily resulting from the presence of heavy metals, dyes, and pharmaceutical residues, including non-steroidal anti-inflammatory drugs (NSAIDs), in aquatic systems [1,2]. The detection of NSAIDs, such as ibuprofen, diclofenac, and ketoprofen, in surface waters and drinking water raises concerns regarding their adverse effects on human health and ecosystems [1,2]. NSAIDs are among the most widely utilized pharmaceuticals globally, entering the environment through point and diffuse sources, including hospital and medical wastewater [3,4]. Typically, they are found in the environment at very low concentrations (ng/L to μg/L), which can lead to harmful ecotoxicological effects even at minimal levels [3,4]. Among NSAIDs, ibuprofen, diclofenac, naproxen, and acetaminophen are the most frequently detected drugs in aquatic environments, resulting in their inclusion on the European Union’s list of ten priority substances for monitoring [3,4].

Effectively addressing this challenge requires the development of efficient and sustainable methods for removing various contaminants, including pharmaceuticals, from water. Such methods encompass electrochemical processes, sonochemical processes, photocatalytic degradation, microextraction, biofiltration, and oxidation [5,6]. Adsorption has emerged as a preferred water purification method due to its simplicity, low cost, and high effectiveness in removing a wide range of contaminants. However, conventional adsorbents often exhibit limitations, including low regeneration capacity, restricted adsorption capacity, and the risk of secondary contamination [4,7,8].

Adsorbents utilized for the removal of NSAIDs from water comprise a diverse array of materials, including activated carbon, biopolymers, metallic nanoparticles, zeolites, ligninolytic enzymes, graphene-based adsorbents, natural polymers, and synthetic polymers such as molecularly imprinted polymers [5,6,9,10].

Recent years have seen promising results from composites based on natural polymers, such as alginate and cellulose, which demonstrate effective adsorption properties, facilitating the removal of not only heavy metals but also organic pollutants, including pharmaceuticals, through the synergistic action of their constituents [11]. Hydrogels derived from natural polymers represent an attractive wastewater and water treatment method, offering numerous advantages such as high safety levels, biodegradability, and low production costs [12]. Their ability to absorb large quantities of water enables the efficient removal of dissolved pollutants. Additionally, their surfaces can be chemically modified, significantly enhancing their adsorption capacity for various environmental contaminants, rendering them particularly useful in advanced purification technologies [12].

Alginate, a natural polysaccharide derived from brown algae, is valued for its gelling properties and ability to form stable structures, making it effective in chelating or adsorbing heavy metal ions such as Pb(II), Cu(II), and Cr(VI), supported by its abundant oxygen-containing functional groups that also facilitate the adsorption of organic compounds [13,14,15]. Cellulose, the most abundant biopolymer, is characterized by mechanical stability and biocompatibility. In its raw form, cellulose exhibits limited adsorption capacity. However, chemical modifications, such as oxidation or the introduction of amino groups, significantly enhance its adsorption capacity for metal ions and organic pollutants [12]. The combination of cellulose with alginate and activated carbon allows for the formation of a porous composite or nanocomposite structure, maximizing the contact surface area with contaminants such as NSAIDs. The enrichment of the cellulose–carbon composite with activated carbon enhances stability and adsorption efficiency due to the high active surface area and the abundance of binding sites [6,16]. Activated carbon, known for its high specific surface area, is commonly used to remove a wide spectrum of pollutants. Its incorporation into the composite increases the capacity to adsorb organic contaminants and provides greater structural stability [6,16]. Furthermore, activated carbon acts as a structural enhancer, rendering the composite durable and reusable [6,16].

Composites based on natural polymers, such as alginate and cellulose, further reinforced with activated carbon, exhibit exceptional physicochemical properties that facilitate the removal of contaminants through appropriate structural modifications, enhancing the selectivity and stability of NSAID adsorption while maintaining low costs and environmental friendliness, which is crucial for environmental applications [17,18].

The aim of this work was to obtain and investigate selected physicochemical and adsorption properties of alginate-based biomaterials. Alginate–cellulose (AlgCa + Cel) and alginate–carbon (AlgCa + C) composites were obtained. Their textural parameters were determined based on low-temperature measurements of nitrogen adsorption and desorption isotherms. The morphology of the samples was assessed using scanning electron microscopy (SEM). Thermal stability was determined based on thermal analysis studies in a synthetic air atmosphere. The degree of swelling of the obtained hydrogels was also determined using the gravimetric method. The acid–base properties of the biocomposites were estimated using the pH drift method. The presence of individual surface functional groups was determined from X-ray photoelectron spectroscopy measurements. The adsorption properties of the biocomposites were also examined. For this purpose, adsorption equilibrium measurements were performed for sodium salts of ibuprofen, diclofenac, and naproxen. The obtained adsorption isotherms were analyzed using the Marczewski–Jaroniec isotherm equation (known as the generalized Langmuir isotherm (GL)). The adsorption kinetics of the tested drugs on the alginate–carbon composite were also examined. The sorption rate data were analyzed using simple adsorption kinetic equations, including first-order (FOE), second-order (SOE), 1,2-mixed-order (MOE), fractal FOE (f-FOE), fractal SOE (f-SOE), fractal MOE (f-MOE), and multi-exponential (m-exp) equations. It is also worth noting that the kinetic data contain almost 100 experimental points. Finally, the literature lacks comprehensive studies on the equilibrium and kinetics of the adsorption of three popularly used non-steroidal anti-inflammatory drugs on alginate composites.

## 2. Materials and Methods

### 2.1. Chemicals

For the synthesis of alginate materials, the following materials were used: sodium alginate (AlgNa; Fluka, London, UK), calcium chloride (CaCl_2_; Sigma-Aldrich, Tokyo, Japan), activated carbon Norit B Supra EUR (C; Norit, Amersfoort, The Netherlands), and microcrystalline cellulose (Cel; Merck, Darmstadt, Germany).

To determine the adsorption properties of alginate composites, sodium salts of the following non-steroidal anti-inflammatory drugs were used as adsorbates: ibuprofen (IBP; Fluka, Mumbai, India), diclofena (D; Sigma, Dongguan City, China), and naproxen (NPX; Sigma, China). The purity of the adsorbates used was ≥98% for IBP and D and 98–100% for NPX. The selected physicochemical properties of these adsorbates are summarized in Table 1.

### 2.2. Material Preparation

A number of alginate materials were obtained by the gelation method: calcium alginate (reference material; AlgCa) and alginate biocomposites with activated carbon (AlgCa + C) and cellulose (AlgCa + Cel).

For this purpose, aqueous solutions of calcium chloride (0.075 mol/L) and sodium alginate (8 g/L), as well as activated carbon and cellulose powder, were used. AlgNa (0.15 L) or its suspension with carbon/cellulose (0.15 L AlgNa + 8 g C/Cel + 0.05 L H_2_O) was added dropwise to an Erlenmeyer flask containing a gelling bath of CaCl_2_ (0.4 L) using a burette at room temperature. The system was stirred using a magnetic stirrer at a speed of 350 rpm. Then, the flask was tightly closed and left for 24 h to completely gel. The obtained hydrogels were filtered and washed with redistilled water. In the final stage, the composite grains were dried in Petri dishes at ambient temperature.

### 2.3. Methodology

#### 2.3.1. Nitrogen Adsorption/Desorption Tests

Nitrogen adsorption and desorption studies for activated carbon, microcrystalline cellulose, and alginate–carbon and alginate–cellulose composites were carried out using the ASAP 2020 surface and porosity analyzer (Micromeritics, Norcross, GA, USA). Before measurements were taken, samples of 0.1 g were degassed at 60 °C. Nitrogen adsorption and desorption of isotherms were performed in the relative pressure p/p_o_ range from 10^−3^ to 0.99 at −196 °C. On their basis, selected textural parameters were calculated: specific surface area (S_BET_), external surface area (S_ext_), total pore volume (V_t_; at p/p_o_ ~ 0.98), including the volume of micropores (V_mic_; t-plot method) and mesopores (V_mes_), and mean hydraulic pore size (D_h_ = 4V_t_/S_BET_).

#### 2.3.2. SEM

SEM micrographs of C, Cel, AlgCa, AlgCa + C, and AlgCa + Cel were taken using a Quanta 3D FEG high-resolution scanning electron-ion microscope (FEI, Ocala, FL, USA) operating at a voltage of 5 kV. Before micrographs were taken, the sample was placed on an aluminum stub and sputtered with gold.

#### 2.3.3. XPS

X-ray photoelectron spectroscopy (XPS) studies were performed for C, Cel, AlgNa, AlgCa, AlgCa + C, and AlgCa + Cel. For this purpose, the tested materials (on a molybdenum carrier) were placed in the loading lock of the ultra-high vacuum system (Prevac) and degassed to obtain a pressure of 7.5 × 10^−8^ mbar (at 25 °C). Then, the sample was transferred to the XPS analytical station, and measurements were taken (7.5 × 10^−9^ mbar). The objects under study were excited with a VG Scienta SAX 100 X-ray tube (Scienta Omicron, Uppsala, Sweden)with an aluminum anode emitting radiation with a specific Kα-Al line of 1486.6 eV. The radiation bandwidth was 0.2 eV, which was possible by using the Monochromator VG Scienta XM 780. CasaXPS software (version 2.3.23) was used for data processing analysis and curve fitting with Shirley-type background ablation.

#### 2.3.4. Thermal Analysis

The thermal stability of C, Cel, AlgCa, AlgCa + C, and AlgCa + Cel was tested in the temperature range from 30 to 950 °C. The experiment was performed using a thermal analysis apparatus equipped with QMS 403D Aelos, STA449F1 Jupiter mass spectrometer (Netzsch, Selb, Germany), and TGA-IR Tensor 27 (Bruker, Billerica, MA, USA). Samples of 15 mg were heated at a rate of 10 °C/min. The measurements were performed in a synthetic air atmosphere (at a flow rate of 25 mL/min).

#### 2.3.5. pH Drift Method

The pH drift method was used to determine the zero charge point of AlgCa + C and AlgCa + Cel. For this purpose, a series of potassium chloride solutions (0.3 L; 0.01 M) with pH ranging from 3 to 12 were prepared. The initial pH_0_ values were adjusted by adding small amounts of hydrochloric acid and sodium hydroxide. Weighted portions (0.3 g) of biocomposites were added to the prepared solutions. The systems were shaken (New Brunswick Scientific Shaker, Edison, NJ, USA) overnight at 25 °C. Finally, the equilibrium pH_r_ values were measured. The zero charge point was determined at the intersection of the ΔpH = f(pH_r_) (ΔpH = pH_r_ − pH_0_) graph with the y-axis.

#### 2.3.6. Swelling Study

For this purpose, biocomposite grains of known mass were immersed in redistilled water and then weighed at regular intervals. Before weighing the swelling granules, excess liquid was removed from their surface. After weighing, the samples were placed in the appropriate solution and left until the next weighing process. The procedure was repeated until equilibrium was achieved, i.e., the maximum swelling of the beads. Then, the swelling ratio (SR) was calculated, which indicates the amount of water absorbed by one gram of composite. This parameter can be calculated using the following formula:

(1) $$\%SR=\frac{m_{i}-m_{0}}{m_{0}}\times 100\%$$

where m_i_ [g]—the mass of the gel after water absorption in equilibrium; m_0_ [g]—the mass of the gel before the absorption process [20].

#### 2.3.7. Adsorption Equilibrium

Adsorption equilibrium investigations of sodium salts of ibuprofen, diclofenac, and naproxen on alginate–carbon and alginate–cellulose composites were performed. The studies were conducted in Erlenmeyer flasks at 25 °C. The initial adsorbate solutions (10 mmol/L) were obtained by dissolving the drug salts in deionized water. The adsorption systems (m = 0.05 g, V = 100 mL, pH = 7) were shaken in a shaker incubated at 110 rpm for a week. Spectrophotometric measurements (Cary 4000 UV–Vis spectrophotometer; Varian, Mulgrave, VIC, Australia) were conducted in the wavelength range of 200–450 nm.

The obtained adsorption isotherms were fitted using the Marczewski–Jaroniec (M-J) isotherm, popularly called the generalized Langmuir adsorption isotherm (GL). It describes adsorption from solutions onto heterogeneous solids and has the following form:

(2) $$\theta ={\left[\frac{{\left(\bar{K}\cdot c_{eq}\right)}^{n}}{{1+\left(\bar{K}\cdot c_{eq}\right)}^{n}}\right]}^{\frac{m}{n}}$$

where Ɵ—surface coverage; Ɵ = a/a_m_; m and n—heterogeneity parameters; and K—the adsorption equilibrium constant. It is worth noting that the Marczewski–Jaroniec isotherm can be transformed into the Langmuir (L; m = n = 1) isotherm equation, the generalized Freundlich (GF; n = 1), Langmuir–Freundlich (LF; m = n), and Tóth (T; m = 1) equations [21,22].

#### 2.3.8. Adsorption Kinetics

Measurements of the adsorption kinetics of ibuprofen sodium, diclofenac sodium, and naproxen sodium were performed on alginate–carbon biocomposite. The initial concentration of adsorbates was 0.298 mmol/L, and the ratio of the composite mass to the volume of the IBP, D, and NPX solution was 1 g/L. Adsorption was carried out in a thermostated vessel (25 °C), the contents of which were mixed with a mechanical stirrer (110 rpm). Spectrophotometric measurements (Cary 100 UV–Vis spectrophotometer; Varian, Australia) were conducted in the wavelength range of 200–450 nm.

The obtained kinetic curves were fitted using simple equations. The first of them was the first-order kinetics equation (FOE/PFOE), which has the following form:

(3) $$ln\left(a_{eq}-a\right)=lna_{eq}-k_{1}t$$

or

(4) $$c=c_{eq}+(c_{o}-c_{eq})\exp(-k_{1}t)$$

where a [mmol/g] is the adsorption amount, indices “o” and “eq” denote the initial and equilibrium values, and k_1_ [s^−1^] represent the coefficient adsorption rates [23,24].

The next equation was the second-order kinetics equation (SOE/PSOE) [24,25,26]:

(5) $$a=a_{eq}\frac{k_{2}t}{1+k_{2}t}$$

where k_2a_ is the rate coefficient of pseudo-second-order kinetics and k_2_ = k_2a_a_eq_ [mol·dm^−3^·s^−1^] (coefficient of second-order kinetics).

Furthermore, the 1,2-mixed-order kinetics equation (MOE) was used. It can be expressed as follows:

(6) $$F=a/a_{eq}=\frac{1-\exp(-k_{1}t)}{1-f_{2}\exp(-k_{1}t)}$$

where f_2_ < 1—the normalized contribution to the kinetics of the second-order process; k_1_—the kinetic coefficient for the partial first-order process [27,28,29].

Additionally, the fractal equivalent of the MOE equation, the fractal-like MOE equation (f-MOE), was also used. It has the following form:

(7) $$F=\frac{{1-exp\left(-k_{1}t\right)}^{p}}{{1-f_{2}exp\left(-k_{1}t\right)}^{p}}$$

where p—the fractal coefficient. It can simplify to the following equations: MOE (p = 0), f-FOE (f_2_ = 0), and f-SOE (f_2_ = 1) [29,30].

However, the best quality of fit was obtained using the multi-exponential (m-exp) equation describing a series of parallel first-order or successive processes. It is expressed as

(8) $$c=(c_{o}-c_{eq})\sum_{i=1}^{n}f_{i}\exp(-k_{i}t)+c_{eq}$$

where f_i_ is the normalized share in the kinetics of process, “i”; k_i_ is the rate coefficient, and u_eq_ = 1 − c_eq_/c_o_ relative to the loss of adsorbate from the solution [25].

## 3. Results

### 3.1. Nitrogen Adsorption/Desorption Studies

**Figure 1:**
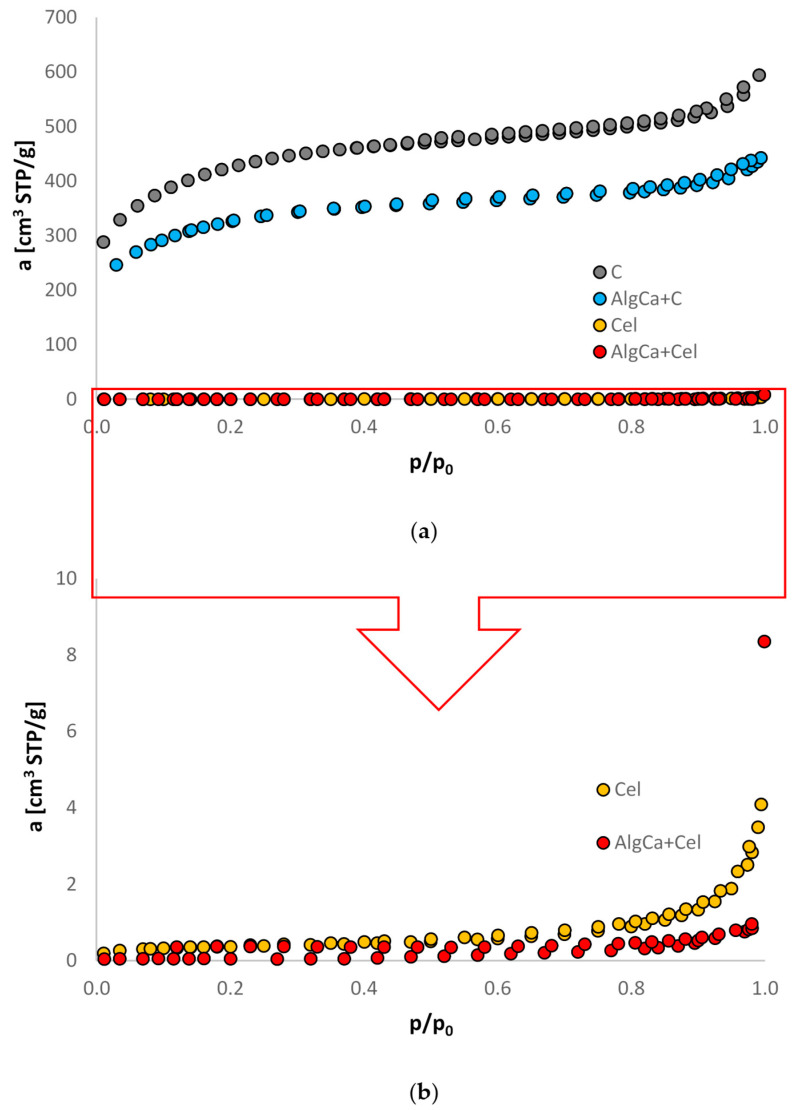
Nitrogen adsorption/desorption isotherms of C (**a**), AlgCa + C (**a**), Cel (**a**,**b**), and AlgCa + Cel (**a**,**b**).

Figure 1 shows low-temperature nitrogen adsorption and desorption isotherms for alginate–carbon and alginate–cellulose biocomposites, and for comparative purposes, they are also shown for pure microcrystalline cellulose and activated carbon. As can be seen, among the composite materials tested, greater adsorption was observed for the AlgCa + C material. Moreover, the shape of the isotherms for the pure activated carbon and its alginate biocomposite, according to IUPAC, classifies them as type I, which is characteristic of microporous adsorbents. It should also be added that they show a small high-pressure hysteresis cycle, which is consistent with the presence of large micropores with a small amount of small mesopores. On the other hand, the shape of the nitrogen adsorption/desorption isotherms for the alginate–cellulose biocomposite as well as for pure microcrystalline cellulose classifies it as type II according to IUPAC, which refers to nonporous materials [31,32].

Table 2 summarizes selected textural parameters for C, Cel, ALgCa + C, and ALgCa + Cel. A clear differentiation of the structure of the obtained biocomposites was observed. The alginate–carbon composite, in relation to the alginate–cellulose composite, was characterized by a highly developed structure, which translated into a large specific surface area and large pore volumes with a significant share of micro- and mesopores. Of course, such spectacular differences result from the differentiation of the properties of activated carbon and cellulose added together with sodium alginate to the gelling bath. Therefore, the values of the selected textural parameters of the biocomposites were similar (due to incorporation in the hydrogel) in relation to their starting materials.

### 3.2. SEM

**Figure 2:**
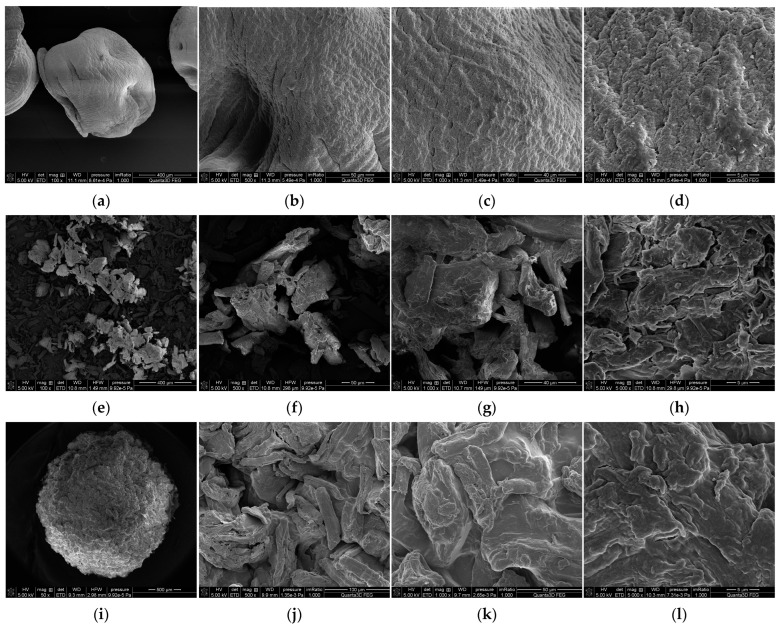

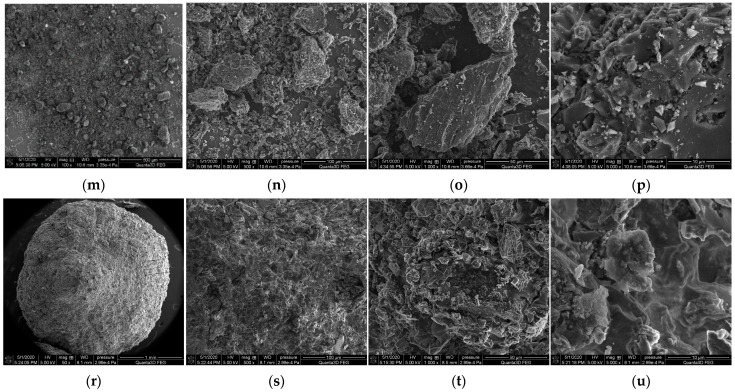
SEM micrographs of ALGCa [magnifications: 100—(**a**); 500—(**b**); 1000—(**c**); 5000—(**d**)], Cel [magnifications: 100—(**e**); 500—(**f**); 1000—(**g**); 5000—(**h**)], AlgCa + Cel [magnifications: 50—(**i**); 500—(**j**); 1000—(**k**); 5000—(**l**)], C [magnifications: 100—(**m**); 500—(**n**); 1000—(**o**); 5000—(**p**)], and AlgCa + C [magnifications: 50—(**r**); 500—(**s**); 1000—(**t**); 5000—(**u**)].

The grain topography of pure calcium alginate and biopolymer incorporated with microcrystalline cellulose and activated carbon was studied using scanning electron microscopy. Figure 2 presents SEM micrographs of AlgCa (Figure 2a–d), Cel (Figure 2e–h), AlgCa + Cel (Figure 2i–l), C (Figure 2m–p), and AlgCa + C (Figure 2r–u). As can be seen, the grains of all alginate materials were spherical. Moreover, the surface of pure AlgCa was practically smooth, and in the case of biocomposites, it was porous. Of course, a more developed structure (from composites) was observed for AlgCa + C. Moreover, it was noted that the materials incorporated into the biopolymer (microcrystalline cellulose and activated carbon) were dusty, and their grains had an irregular shape. The above data are in accordance with the data obtained from nitrogen sorption measurements.

### 3.3. XPS

**Figure 3:**
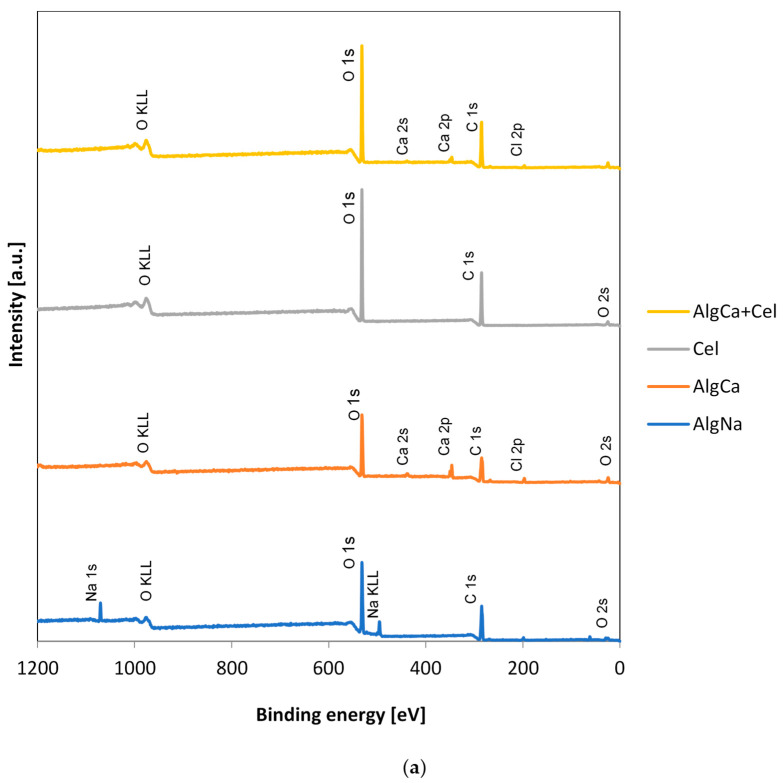

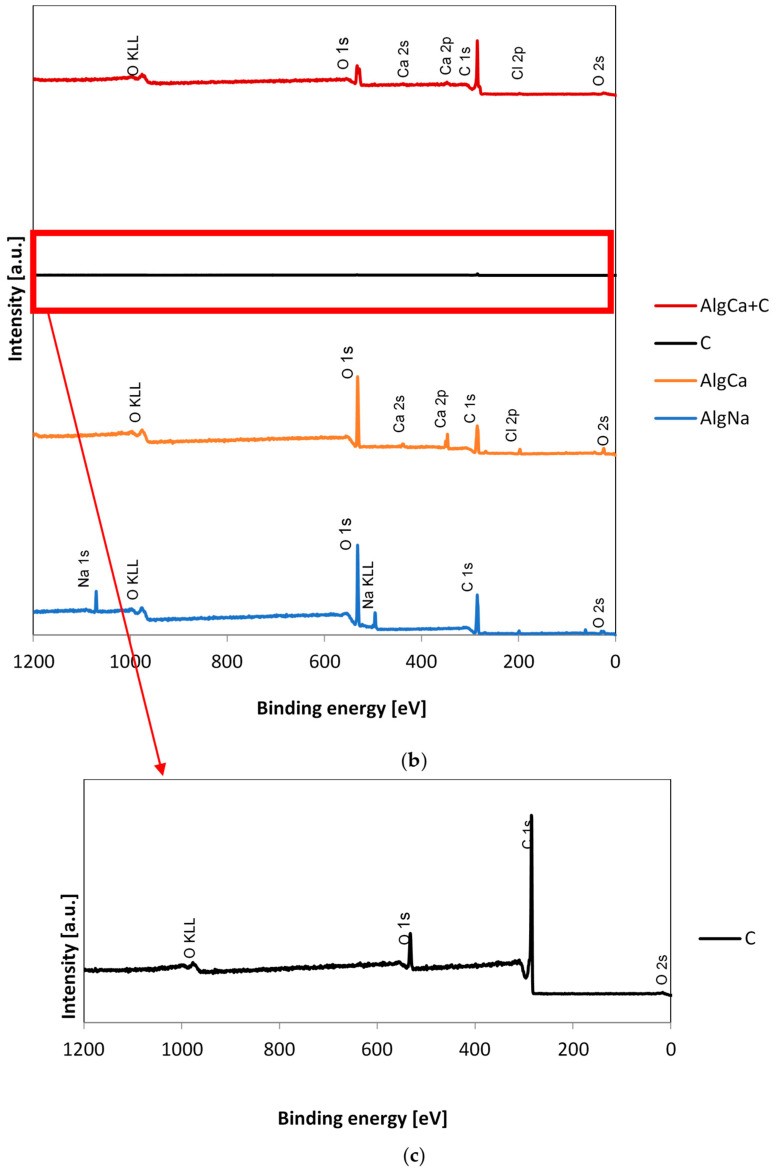
XPS survey spectra of AlgNa (**a**,**b**), AlgCa (**a**,**b**), Cel (**a**), C (**b**,**c**), AlgCa + Cel (**a**), and AlgCa + C (**b**).

Measurements were carried out using X-ray photoelectron spectroscopy for the analysis of the elemental composition and chemical binding for alginate materials (ALgCa, AlgCa + Cel, and AlgCa + C) and their starting materials (AlgNa, Cel, and C). The obtained results are shown in Figure 3 and Figures S1–S6 and in Table 3 and Table 4. Figure 3 shows the general XPS spectra for AlgNa (Figure 3a,b), AlgCa (Figure 3a,b), Cel (Figure 3a,b), AlgCa + Cel (Figure 3a), C (Figure 3b,c), and AlgCa + C (Figure 3b). For all the samples studied, distinct bands with binding energies of ~285 eV and ~532 eV were noted, corresponding to the presence of carbon C 1s and oxygen O 1s, respectively. Additionally, for all obtained hydrogels, bands with binding energies of ~347 eV and 197 eV were observed, indicating the presence of calcium Ca 2p and chlorine Cl 2p, respectively. For sodium alginate, a band with a binding energy of ~1070 eV was also recorded, corresponding to the presence of sodium Na 1s.

The atomic concentrations of the investigated elements were also determined: C 1s: 64.4 at.%, O s1: 33.3 at.%, and Na 1s: 2.3 at.% for sodium alginate; C 1s: 57.4 at.%, O s1: 33.9 at.%, Ca 2p: 4.3 at.%, and Cl 2p: 3.0 at.% for calcium alginate; C 1s: 59.2 at.% and O s1: 40.8 at.% for cellulose; C 1s: 95.1 at.% and O s1: 4.9 at.% for activated carbon; C 1s: 57.2 at.%, O s1: 40.1 at.%, Ca 2p: 1.4 at.%, and Cl 2p: 1.3 at.% for alginate–cellulose composite; and C 1s: 78.3 at.%, O s1: 19.5 at.%, Ca 2p: 0.7 at.%, and Cl 2p: 1.5 at.% for the alginate–carbon composite (Table 3).

In the next part of the experiment, detailed XPS spectra were obtained for carbon C 1s, oxygen O 1s, and calcium Ca 2p. This allowed for the determination of individual chemical groups on the surface of alginate hydrogels and their starting raw materials. The obtained data are summarized in Figures S1–S6 and in Table 4.

The analysis of the data presented in Figures S1a and S2a and in Table 3 showed that the carbon lines for AlgNa and AlgCa are distributed into four singlet spectral lines. The first one at a BE of 285 eV corresponds to a single C-C bond between carbon atoms and a carbon–hydrogen bond (C-H). The next line, with a binding energy of 286.6 eV, indicates the occurrence of a single bond between carbon and the oxygen of a hydroxyl group (C-OH) or the oxygen of an ether group (C-O-C). Another line, at a BE of 288.1 eV, indicates the presence of a single bond of carbon to two oxygen atoms (O-C-O). The last line, at a bond energy of 289.3 eV, corresponds to carbon in an ester group (COOR).

In the case of cellulose and the alginate–cellulose composite (Figures S3a and S4a, Table 3), the carbon lines are also distributed into four spectral lines. Two of them (BE of 285 eV and 286.8 eV) are analogous to those for sodium and calcium alginates. The next line was recorded at a binding energy of 288.1 eV, which means there was a single bond between the carbon and oxygen of the hydroxyl group C-OH. The last line, at a BE of 289.1 eV, corresponds to the carbon from the carboxyl group -COOH.

For activated carbon and the alginate–carbon biocomposite (Figures S5a and S6a, Table 3), the carbon lines are distributed into seven spectral lines. The obtained data indicate the presence of carbon in the following chemical groups: C-H sp^3^ (BE of 285 eV), C=C sp^2^ (BE of 284.53 eV), C-C sp^3^ (BE of 285.67 eV), C-OH (BE of 286.32 eV), C-O-C (BE of 287.13 eV), C=O (BE of 288.24 eV), and C=O (BE of 288.24 eV) for carbon and C=C sp^2^ (BE of 284.4 eV), C-OH (BE of 286.5 eV), C=O (BE of 288.0 eV), COOR (BE of 289.2 eV), C-C/C-H (BE of 278.9 eV), C-OH/C-O-C (BE of 288.24 eV), O-C-O (BE of 282.2 eV), and COOR (BE of 283.2 eV) for AlgCa + C.

The analysis of the data presented in Figures S1b and S2b and in Table 3 showed that the oxygen lines for AlgNa and AlgCa are distributed into three singlet spectral lines. The first one (BE of 531.4 eV) corresponds to the oxygen forming a double bond with the carbon of the ester group, O=C-O-R, and the single bond of oxygen with sodium (O-Na) (for AlgNa) or calcium (O-Ca) (for AlgCa). The next line (BE of 532.9 eV) corresponds to the oxygen of the hydroxyl group (C-OH). The last line, with a binding energy of 533.4 eV, corresponds to an oxygen atom joined by single bonds to two carbon atoms (C-O-C) and oxygen forming single bonds in the ester group COOR.

For cellulose (Figure S3b and Table 3), oxygen is distributed into two singlet lines. The first one, with a binding energy of 532.9 eV, corresponds to a single bond of oxygen from the hydroxyl group with a carbon atom (C-OH). The second one, with a BE of 533.5 eV, indicates the presence of an oxygen atom joined by single bonds to two carbon atoms (C-O-C).

For the AlgCa + Cel biocomposite (Figure S4b and Table 3), oxygen was observed to distribute into three spectral lines. Two of them (BE of 533 eV and 533.6 eV) are analogous to those for cellulose. The third, with a binding energy of 531.6 eV, corresponds to the oxygen forming a double bond with the carbon of the ester group O=C-O-R and the single bond of oxygen with calcium (O-Ca).

In the case of activated carbon (Figure S5b and Table 3), oxygen is distributed over five spectral lines. They indicate the presence of this element in the following chemical groups: O^−2^, O=<=>=O (BE of 530.3 eV); O=C-O, O=C (BE of 531.43 eV); C-O, O=C (BE of 532.68 eV); OH-C, O-C=O (BE of 533.75 eV); and H_2_O/O_2_ (BE of 535.40 eV).

Figures S2c, S4c and S6c and Table 3 show that the calcium lines distribute into two spectral lines. They occur at binding energies of 347.9 eV and 351.5 eV, corresponding to the occurrence of calcium Ca 2p 3/2 and Ca 2p 1/2, respectively.

It should also be noted that for the alginate–carbon composite, individual spectral lines for oxygen (Figure S6b) and calcium (Figure S6c) atoms were not distinguished. The peaks originating from O 1s and Ca 2p atoms occurred in places with conductive carbon, which made it impossible to identify the chemical forms in detail.

### 3.4. Thermal Analysis

**Figure 4:**
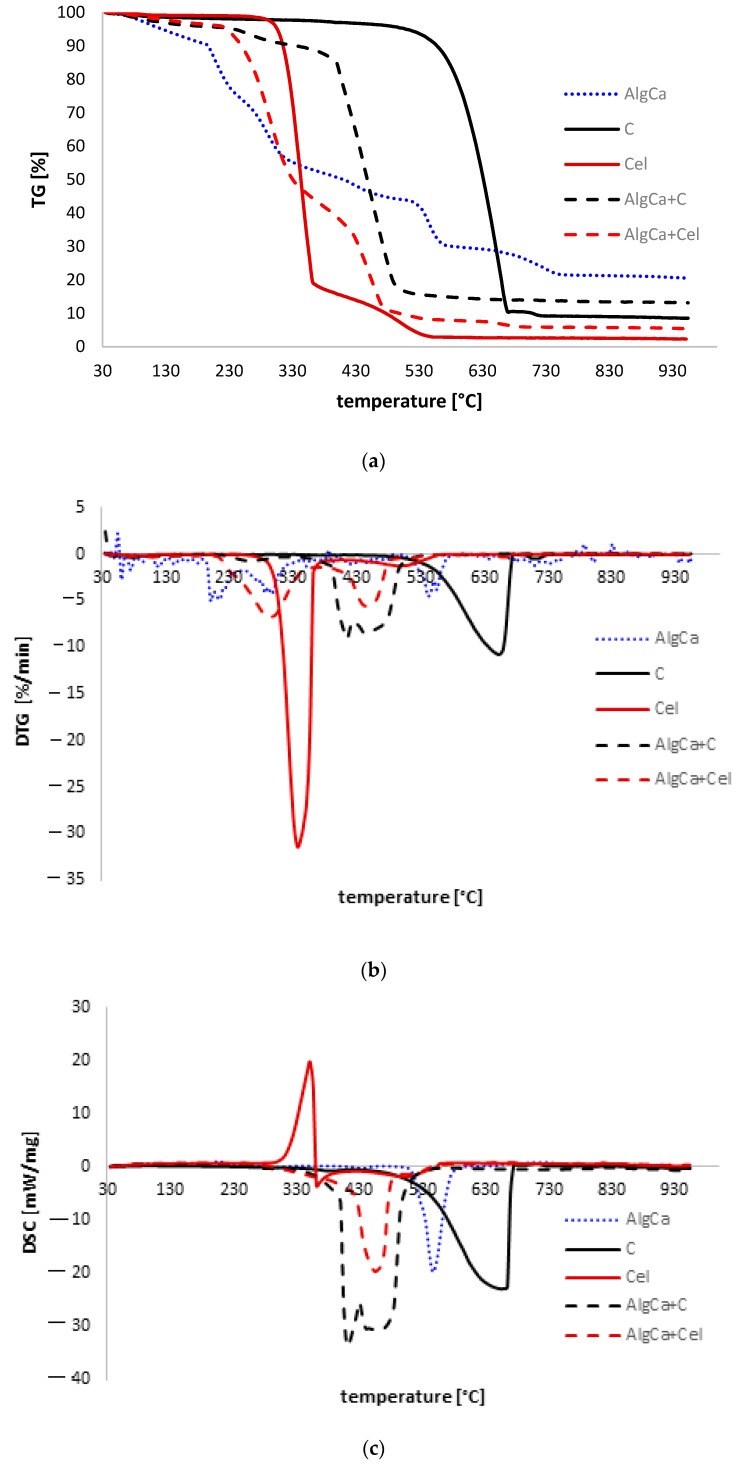
Comparison of TG (**a**), DTG (**b**), and DSC (**c**) curves of AlgNa, AlgCa, Cel, C, AlgCa + Cel, and AlgCa + C.

The thermal stability of the obtained alginate hydrogels and, for comparative purposes, microcrystalline cellulose and activated carbon, was also tested. Figure 4 presents a comparison of the TG (Figure 4a), DTG (Figure 4b), and DSC (Figure 4c) curves for C, Cel, AlgCa, AlgCa + C, and AlgCa + Cel. Table S1 shows data determined on the basis of these curves (TG, DTG, and DSC).

For all tested samples, two distinct stages of the decomposition process were noted. Among the tested hydrogels, the smallest mass loss was recorded for calcium alginate (79.45%), and the largest for the alginate–cellulose biocomposite (94.51). The first stage of decomposition (endothermic process) took place in the temperature range from 30 °C to 200 °C with losses of 11.23%, 4.31%, and 3.79% for AlgCa, AlgCa + C, and AlgCa + Cel, respectively. The desorption of physically adsorbed water and the removal of surface hydroxyl groups take place here. The second stage, in the temperature range from 200 °C to 950 °C, occurs with mass losses of 68.22% (AlgCa), 87.16% (AlgCa + C), and 90.72% (AlgCa + Cel) and corresponds to the destruction and combustion of the hydrogels (exothermic process). It should be added that drastic weight losses for pure calcium alginate occur at temperatures of 215.3 °C, 306.5 °C, and 545.3 °C. In the case of the alginate–cellulose biocomposite, significant weight losses were recorded at 294.9 °C and 444.8 °C. Finally, for the alginate–carbon composite, it was observed that the main oxidation process occurs at a temperature of approximately 430.5 °C.

It should also be noted that the TG for alginate biocomposites, in relation to pure cellulose and activated carbon, have a mild shape, as a result of which there is no sharp temperature change boundary, especially for AlgCa + Cel. This suggests that the obtained hydrogels contain an amorphous phase, which makes them more flexible.

### 3.5. pH Drift Method

**Figure 5:**
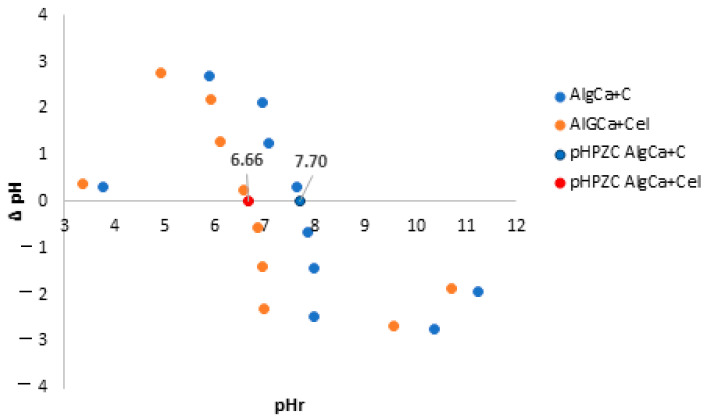
Point of zero charge for AlgCa + Cel and AlgCa + C.

In order to estimate the acid–base properties of the obtained alginate biocomposites, the zero charge point pH_PZC_ was estimated by the pH drift method, and the results are presented in Figure 5. As can be seen, the alginate–cellulose composite was slightly acidic (pH_PZC_ = 6.66), and the alginate–carbon material was slightly alkaline (pH_PZC_ = 7.70).

### 3.6. Determination of Swelling Index

**Figure 6:**
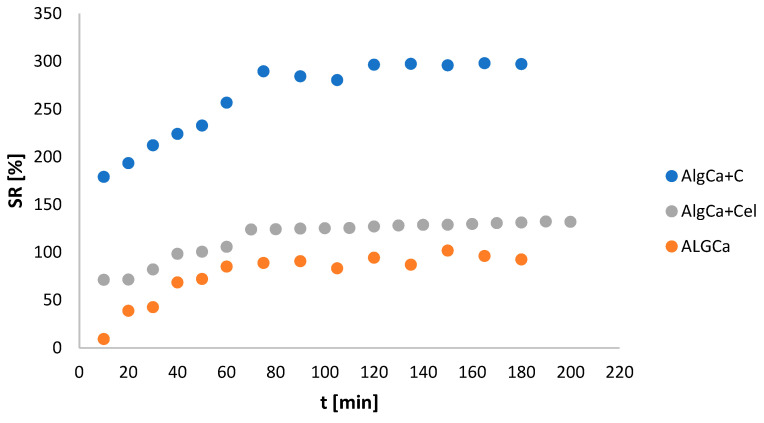
Dependence of swelling index on incubation time in redistilled water for AlgCa, AlgCa + Cel, and AlgCa + C.

One of the important properties of hydrogels is their ability to swell. In connection with the above, it was reasonable to determine the swelling degree (SR) of the obtained calcium alginate and its biocomposites with microcrystalline cellulose and activated carbon. As can be seen in Figure 6, for all tested materials, the maximum swelling degree was achieved after about 75 min. Moreover, higher SR values were observed for biocomposites in relation to pure AlgCa. Additionally, for AlgCa + C, a swelling coefficient close to 300% was achieved, and for AlgCa + Cel, this value was about 130%, while for calcium alginate, the SR was at the level of up to 100%. 

### 3.7. Adsorption Equilibrium

**Figure 7:**
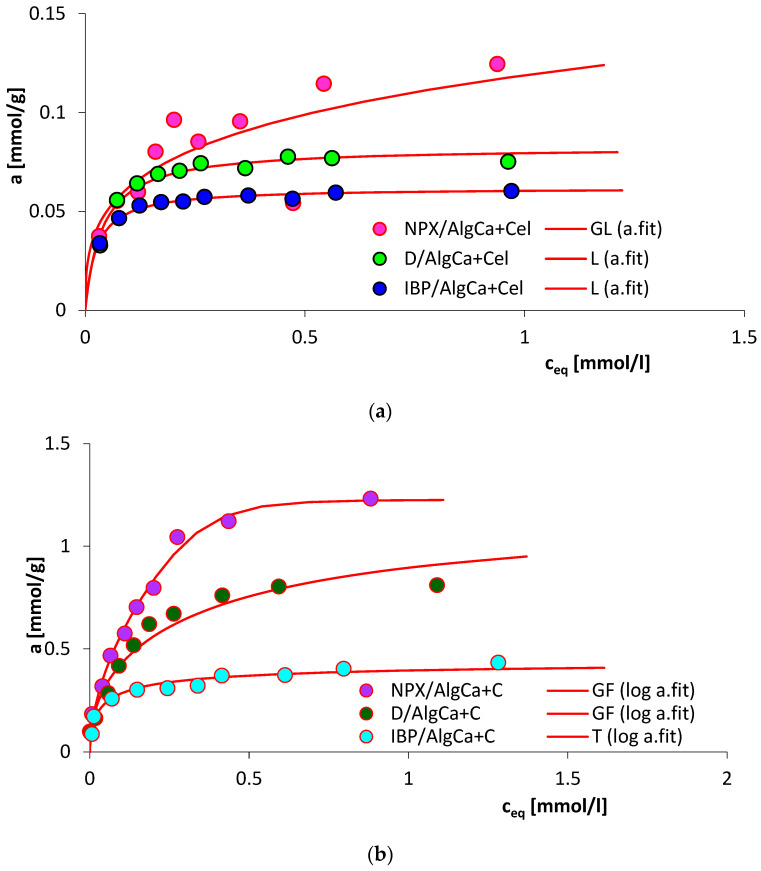
The adsorption isotherms for IBP, D, and NPX on AlgCa + Cel (**a**) and AlgCa + C (**b**).

Figure 7 compares the adsorption isotherms of ibuprofen sodium, diclofenac sodium, and naproxen sodium on alginate–cellulose (Figure 7a) and alginate–carbon (Figure 7b) bicomposites. As can be seen, significantly higher adsorption values were recorded in the systems with AlgCa + C (1.23 mmol/g for NPX, 0.81 mmol/g for D, and 0.43 mol/g for IBP) than for AlgCa + Cel (0.12 mmol/g for NPX, 0.075 mmol/g for D, and 0.06 mol/g for IBP). The hydrogel incorporated with activated carbon is characterized by a large specific surface area, a large pore volume, and a pore diameter (Dh = 3.76 nm) enabling the penetration of its internal structure by selected adsorbates, which undoubtedly favors adsorption. Moreover, under the given experimental conditions (pH = 7), the alginate–carbon composite (pH_PZC_ = 7.70) had a slight positive charge, and IBP, D, and NPX were present as anions. Therefore, electrostatic attraction interactions may occur, which additionally intensifies the adsorption rate. The alginate–cellulose biocomposite is characterized by a small specific surface area and small pore volume, which does not favor adsorption. Moreover, AlgCa + Cel under the given experimental conditions had a negative charge (pH_PZC_ = 6.66), which could lead to the occurrence of electrostatic repulsion forces, and this could additionally weaken adsorption.

It should be added that the adsorption mechanism of the tested drugs for the alginate–carbon composite systems is also based on the occurrence of dispersion interactions between π electrons from the aromatic rings of the adsorbate molecules and π electrons from the graphene layers of carbon. Moreover, hydrogen interactions occur between the functional groups of IBP, D, and NPX and the surface groups of the biocomposites.

Additionally, in both the alginate–carbon and alginate–cellulose biocomposite systems, the highest adsorption values were noted for sodium naproxen, and the lowest for sodium ibuprofen. This effect is caused by differences in the hydrophilicity and structure of the tested sodium salts of non-steroidal anti-inflammatory drugs. Among the adsorbates used, IBP is characterized by the lowest hydrophobicity (c_s_ = 100 mg/mL) and therefore has the lowest affinity for lyophilic organic adsorbents. On the other hand, D is the most hydrophobic (c_s_ = 50 mg/mL), but at the same time, it is characterized by the largest particle size, which blocks its penetration into the narrow pores of AlgCa + C.

The adsorption isotherms were fitted using the M-J isotherm, and the estimated parameters are listed in Table 5. For the adsorption of sodium naproxen and sodium diclofenac on the alginate–carbon biocomposite, the heterogeneity parameter n is equal to 1, which makes the Marczewski–Jaroniec isotherm become reduced to the generalized Freundlich equation. For the adsorption of sodium ibuprofen on AlgCa + C, the heterogeneity parameter m is equal to 1, which indicates that the M-J isotherm is reduced to the Tóth equation. In the case of the adsorption of IBP and D on the alginate–cellulose biocomposite, the parameters m and n are equal to 1, which means that the Marczewski–Jaroniec isotherm is simplified to the Langmuir isotherm. Only for the NPX/AlgCa + Cel system, the full form of the generalized Langmuir isotherm was used. Moreover, it is worth noting that the obtained fitted values of adsorption capacity are very close to the experimental values. Furthermore, the estimated values of standard deviations (SD(a) from 0.018 to 0.062) and determination coefficients (R^2^ ranging from 0.941 to 0.988) indicated a good quality of fit.

### 3.8. Adsorption Kinetics

Due to the fact that significantly lower adsorption values of sodium salts of non-steroidal anti-inflammatory drugs were obtained for the alginate–cellulose biocomposite, adsorption kinetics studies were conducted for systems with alginate–carbon material.

**Figure 8:**
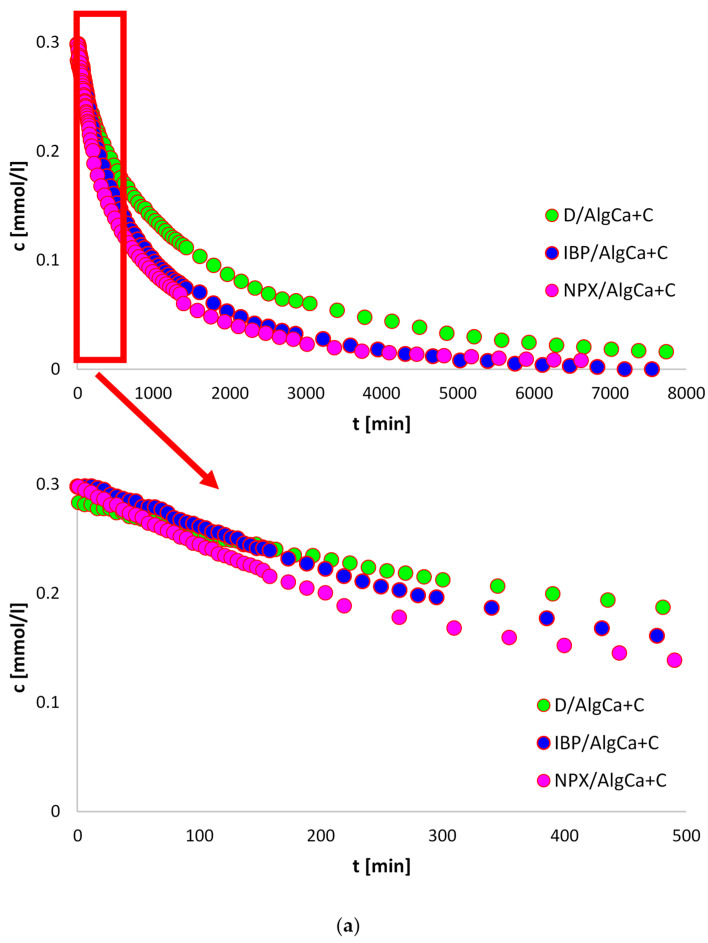

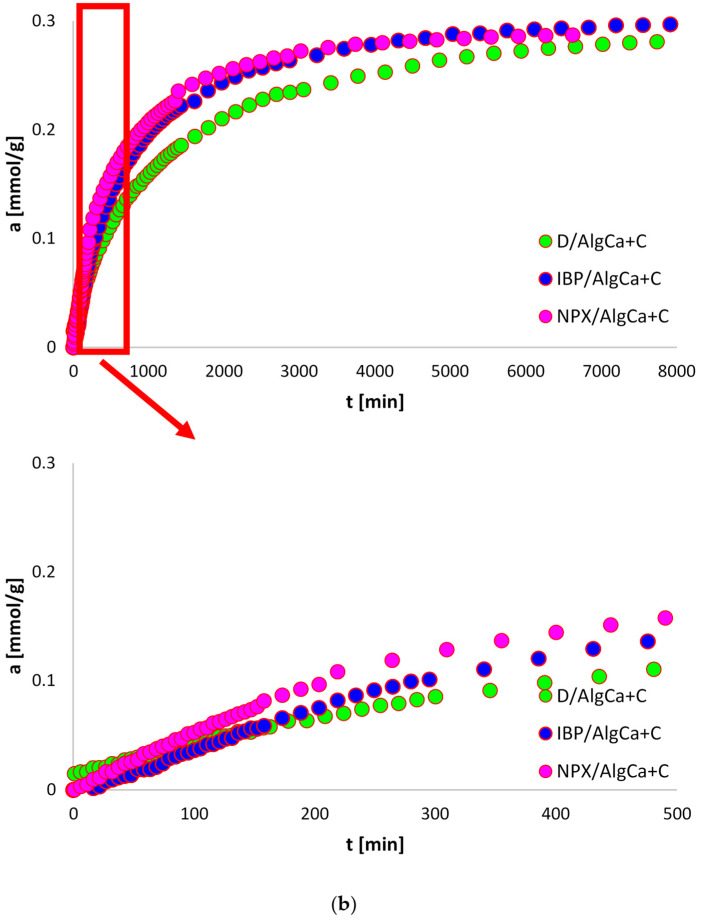
The adsorption kinetics for IBP, D, and NPX on AlgCa + C shown as changes in concentration (**a**) and adsorption (**b**) over time.

Figure 8 compares the kinetic curves for IBP, D, and NPX on AlgCa + C presented as profiles of concentration changes (Figure 8a) and relative adsorption (Figure 8b) over time. As can be seen, the highest adsorption rate was noted for sodium naproxen, and the lowest for sodium diclofenac. NPX is characterized by smaller dimensions in relation to D and lower hydrophilicity in relation to IBP, which caused its adsorption to occur in a shorter time. However, due to their large dimensions, sodium diclofenac molecules diffuse more slowly to the adsorbent surface and, at the same time, may have difficulties in entering narrow pores, which prolongs the adsorption time.

We attempted to fit the obtained adsorption rate data to simple adsorption kinetic equations, the relative standard deviations of which are listed in Table 6. As can be seen, the best fit was obtained using the multi-exponential equation, the parameters of which are listed in Table 7. It was noted that the adsorption of sodium ibuprofen, sodium diclofenac, and sodium naproxen is a complicated process, the behavior of which is correctly described by two terms of the m-exp equation. It is also worth paying attention to the values of the half-times, t_1/2_. In this particular case, where the total loss of drugs from the solutions occurs (u_eq_ = 1), they correspond to the times after which the concentration of the pharmaceutical is 0.5c_0_. The estimated values of the half-times were 415.7 min, 529.8 min, and 842.3 min for NPX, D, and IBP, respectively. Finally, it is worth noting the low values of relative standard deviations (0.335% < SD(c)/co < 0.720%) and low values of uncertainty coefficients (3.9 × 10^−4^ < 1 − R^2^ <6.9 × 10^−4^), which prove that there is a very good quality of fit.

## 4. Discussion

In the presented work, alginate–carbon and alginate–cellulose biocomposites were obtained. It was shown that the hydrogel incorporated with activated carbon showed attractive textural, thermal, and adsorption properties. AlgCa + C was characterized by a high specific surface area of 1151 m^2^/g and high thermal stability (the main process of mass loss at 430 °C). Moreover, it was recorded that it can be an effective sorbent of non-steroidal anti-inflammatory drugs, which translates into sorption capacities of 310.27 mg/g, 257.69 mg/g, and 98.15 mg/g for NPX, D, and IBP, respectively. There are reports in the literature on the preparation of alginate composites, but they are often characterized by significantly lower efficiency in removing non-steroidal anti-inflammatory drugs from aqueous solutions. Moreover, in the presented work, the kinetic curves contain almost 100 experimental points, which indicates the high quality of the experimental data. Finally, the available information most often presents the adsorption of one or two NSAIDs. Table 8 presents an overview of hydrogels of a similar type for the removal of drugs from aqueous solutions.

## 5. Conclusions

The obtained alginate hydrogels were alginate biocomposites incorporated with microcrystalline cellulose and activated carbon and, for comparative purposes, pure calcium alginate. The obtained composites showed different textural, topographic, thermal, acid–base, and adsorption properties. It was shown that AlgCa + C was characterized by a strongly developed texture, which translated into a large specific surface area (1151 m^2^/g) and large pore volumes (0.69 cm^3^/g), with an indication of a significant share of micropores (0.23 cm^3^/g). Additionally, the average hydraulic pore size for the alginate–carbon composite was 3.76 nm. Additionally, the SEM micrographs showed that the surface of the pure hydrogel is smooth, and that of the biocomposites is porous, which is in agreement with the data obtained from the nitrogen adsorption and desorption studies. In a later part of the study, based on the results of the XPS studies, individual chemical groups were identified in the samples. Moreover, the highest % carbon content was recorded for the alginate–carbon biocomposite (78.3%), and the lowest for the alginate–cellulose one (57.2%). Thermal analysis showed that the biocomposites were more thermally stable compared to the pure hydrogel. The mass losses up to 200 °C were 11.23%, 4.31%, and 3.79% for AlgCa, AlgCa + C, and AlgCa + Cel, respectively. Moreover, the shape of thermogravimetric curves for hydrogels is gentle, without sharp temperature steps, which indicates their plasticity. This feature has potential for applications in medicine and pharmacy. The zero charge point was also estimated, showing that the alginate–cellulose (pH_PZC_ = 6.66) composite was slightly acidic and the alginate–carbon (pH_PZC_ = 7.70) composite was alkaline. The swelling degree of the obtained hydrogels was also determined. It was shown that the composites, in relation to pure calcium alginate, were characterized by a greater ability to absorb water. The swelling indices were 100%, 130%, and 300% for AlgCa, AlgCa + Cel, and AlgCa + C, respectively. From a practical point of view, they could be used in cosmetics, pharmacy, medicine, or agriculture. Finally, based on the measurements of adsorption kinetics and equilibrium, the sorption properties of the biocomposites were determined in relation to sodium salts of non-steroidal anti-inflammatory drugs. Among the adsorbents tested, AlgCa + C showed high efficiency. The obtained adsorption values were 1.23 mmol/g for NPX, 0.81 mmol/g for D, and 0.43 mmol/g for IBP. Moreover, it was noted that sodium naproxen was also the fastest to adsorb. The calculated values of the t_1/2_ were 415.7 min, 529.8 min, and 842.3 min for NPX, D, and IBP, respectively. In connection with the above, the alginate–carbon composite can be used in environmental protection as an adsorbent of anthropogenic pollutants.

## Figures and Tables

**Table 1:** Chosen physicochemical properties of IBP, NPX, and D [19].

Adsorbate	Chemical Formula	M ^a^ [g/mol]	c_s_ ^b^ [mg/mL at 25 °C]	pKa ^c^	^d^ m.p. [°C]	^e^ b.p. [°C]	^f^ T.P.S.A. [Å^2^]
IBP	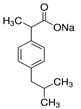	228.26	100	4.91	75–77	319.6	40.1
NPX	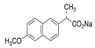	252.24	76	4.15	250–251	403.9	49.4
D	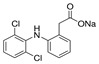	318.13	50	4.15	288–290	412	52.2

^a^ M—molar mass; ^b^ c_s_—solubility in H_2_O at 25 °C; ^c^ pK_a_—ionization constant; ^d^ m.p.—melting point; ^e^ b.p.—boiling point; ^f^ T.P.S.A.—Topological Polar Surface Area.

**Table 2:** Textural parameters of C, Cel, AlgCa + C, and AlgCa + Cel.

Adsorbent	Surface Area [m^2^/g]		Pore Volume [cm^3^/g]		Pore Size [nm]	
	S_BET_ ^a^	S_ext_ ^b^	V_t_ ^c^	V_mes_ ^d^	V_mic_ ^e^	D_h_ ^f^
C	1376	691	0.87	0.21	0.55	2.68
AlgCa + C	1151	709	0.69	0.28	0.23	3.76
Cel	1.35	0.97	0.0065	0.0039	-	19.13
AlgCa + Cel	0.18	0.18	0.0013	-	-	16.97

^a^ S_BET_, BET-specific surface area; ^b^ S_ext_, external surface area; ^c^ V_t_, total pore volume; ^d^ V_mic_, mesopore volume; ^e^ V_mic_, micropore volume; ^f^ D_h_, average hydraulic pore diameter.

**Table 3:** XPS survey spectra results and determined elemental compositions (as atomic%) for AlgNa, AlgCa, Cel, C, AlgCa + Cel, and AlgCa + C.

Sample	Name	Peak Position [eV]	Full Width at Half Maximum (FWHM)	Atomic Concentration [at.%]
AlgNa	C 1s	285.0	3.3	64.4
	O 1s	531.8	2.3	33.3
	Na 1s	1070.3	1.9	2.3
AlgCa	C 1s	285.0	4.3	57.4
	O 1s	531.8	2.8	33.9
	Ca 2p	346.5	2.0	4.3
	Cl 2p	197.3	2.9	3.0
Cel	C 1s	285.0	2.0	59.2
	O 1s	531.8	1.8	40.8
C	C 1s	284.0	2.25	95.1
	O 1s	531.5	3.93	4.9
AlgCa + Cel	C 1s	285.0	2.2	57.2
	O 1s	531.8	2.0	40.1
	Ca 2p	346.5	1.9	1.4
	Cl 2p	196.5	3.1	1.3
AlgCa + C	C 1s	285.0	2.1	78.3
	O 1s	532.5	7.5	19.5
	Ca 2p	347.3	3.5	0.7
	Cl 2p	198.0	2.0	1.5

**Table 4:** C 1s, O 1s, and Ca 2p in various chemical combinations and their atomic concentrations for AlgNa, AlgCa, Cel, C, AlgCa + Cel, and AlgCa + C.

Sample	Name	Peak Position [eV]	Full Width at Half Maximum (FWHM)	Atomic Concentration [at.%]	Suggested Binding
AlgNa	C 1s A	285.0	1.2	26.3	C-C/C-H
	C 1s B	286.6	1.1	30.9	C-OH/C-O-C
	C 1s C	288.1	1.4	12.5	O-C-O
	C 1s D	289.3	1.3	1.3	COOR
	O 1s A	531.4	1.38	21.3	O=C-O-R/O-Na
	O 1s B	532.9	1.35	54.1	C-OH
	O 1s C	533.4	1.35	24.6	C-O-C/O=C-O-R
AlgCa	C 1s A	285.0	1.3	36.2	C-C/C-H
	C 1s B	286.8	1.4	42.3	C-OH/C-O-C
	C 1s C	288.5	1.4	18.4	O-C-O
	C 1s D	289.3	1.3	3.1	COOR
	O 1s A	531.8	1.53	29.6	O=C-O-R/O-Ca
	O 1s B	533.1	1.58	48.5	C-OH
	O 1s C	533.7	1.58	21.9	C-O-C/O=C-O-R
	Ca 2p A	347.9	1.45	52.6	Ca 2p 3/2
	Ca 2p B	351.4	1.48	47.4	Ca 2p 1/2
Cel	C 1s A	285.0	1.3	10.1	C-C/C-H
	C 1s B	286.7	1.1	73.0	C-OH/C-O-C
	C 1s C	288.1	1.0	14.9	C-OH
	C 1s D	289.1	1.4	2.0	COOH
	O 1s A	532.9	1.25	60.0	C-OH
	O 1s B	533.5	1.25	40.0	C-O-C
C	C 1s A	285.00	0.71	8.5	C-H sp3
	C 1s B	284.53	0.66	56.7	C=C sp2
	C 1s C	285.67	0.73	4.1	C-C sp3
	C 1s D	286.32	0.75	3.1	C-OH
	C 1s E	287.13	0.79	1.3	C-O-C
	C 1s F	288.24	0.77	0.2	C=O
	C 1s G	284.10	0.66	26.2	O=C-O-
	O 1s A	530.30	1.34	12.67	O^−2^, O=<=>=O
	O 1s B	531.43	1.32	26.67	O=C-O, O=C
	O 1s C	532.68	1.32	33.03	C-O, O=C
	O 1s D	533.75	1.32	18.95	OH-C, O-C=O
	O 1s E	535.40	1.70	8.68	H_2_O/O_2_
AlgCa + Cel	C 1s A	285.0	1.2	13.9	C-C/C-H
	C 1s B	286.8	1.1	67.9	C-OH/C-O-C
	C 1s C	288.2	1.0	13.9	C-OH
	C 1s D	289.0	1.3	4.3	COOH
	O 1s A	533.0	1.29	54.6	C-OH
	O 1s B	533.6	1.29	36.4	C-O-C
	O 1s C	531.6	1.62	9.0	O-Ca/O=C-O-R
	Ca 2p A	347.9	1.48	50.5	Ca 2p 3/2
	Ca 2p B	351.5	1.50	49.5	Ca 2p 1/2
AlgCa + C	C 1s A	284.4	0.9	66.1	C=C sp2 (carbon)
	C 1s B	286.5	1.5	5.9	C-OH (carbon)
	C 1s C	288.0	1.5	2.7	C=O (carbon)
	C 1s D	289.2	1.5	1.8	COOR (carbon)
	C 1s E	278.9	1.5	7.5	C-C/C-H (alginate)
	C 1s F	280.7	1.5	7.5	C-OH/C-O-C (alginate)
	C 1s G	282.2	1.4	4.2	O-C-O (alginate)
	C 1s H	283.2	1.5	4.3	COOR (alginate)

**Table 5:** Parameters of the Marczewski–Jaroniec equation for the adsorption of ibuprofen sodium, diclofenac sodium, and naproxen sodium on AlgCa + Cel and AlgCa + C biocomposites.

System	Isotherm Type	a_m_ ^a^	m ^b^	n ^b^	logK ^c^	R^2 d^	SD(a) ^e^
NPX/AlgCa + C	GF	1.24	0.51	1	0.47	0.988	0.018
IBP/AlgCa + C	T	0.47	1	0.53	1.96	0.961	0.046
D/AlgCa + C	GF	0.89	0.48	1	0.11	0.975	0.041
NPX/AlgCa + Cel	GL	0.14	0.78	0.54	0.31	0.941	0.043
IBP/AlgCa + Cel	L	0.06	1	1	0.87	0.948	0.062
D/AlgCa + Cel	L	0.08	1	1	0.91	0.962	0.045

^a^ a_m_—sorption capacity; ^b^ m and n—heterogeneity parameters; ^c^ logK—logarithm of adsorption equilibrium; ^d^ R^2^—determination coefficient; ^e^ D(a)—standard deviation.

**Table 6:** Relative standard deviations (SD(c)/c_o_) (%) for m-exp, FOE, SOE, MOE, f-FOE, f-SOE, and f-MOE.

System	m-exp [%]	FOE [%]	SOE [%]	MOE [%]	f-FOE [%]	f-SOE [%]	f-MOE [%]
NPX/AlgCa + C	0.798	2.489	3.105	1.129	0.983	1.984	0.864
IBP/AlgCa + C	0.739	2.991	3.115	0.926	1.041	1.684	1.101
D/AlgCa + C	0.871	5.312	2.009	1.078	0.903	1.742	0.986

**Table 7:** Optimized parameters of m-exp eq.

System	f_1_^a^, log k_1_^b^	f_2_^a^, log k_2_^b^	u_eq_^c^	t_1/2_^d^ [min]	SD(c)/c_o_^e^ [%]	1 − R^2 f^
NPX/AlgCa + C	0.532, −3.14	0.468, −2.48	0.996	415.7	0.798	6.5 × 10^−4^
IBP/AlgCa + C	0.538, −3.26	0.462, −2.58	1	529.8	0.739	3.9 × 10^−4^
D/AlgCa + C	0.719, −3.36	0.281, −2.52	1	842.3	0.871	6.9 × 10^−4^

^a^ f_1_ and f_2_—terms of m-exp equation; ^b^ log k_1_ and log k_2_—logarithms of rate constant; ^c^ u_eq_—relative loss of adsorbate from solution; ^d^ t_1/2_—half-time; ^e^ SD(c)/c_o_—relative standard deviation; ^f^ 1 − R^2^—indetermination coefficient.

**Table 8:** The application of hydrogels in the adsorption of non-steroidal anti-inflammatory drugs.

Adsorbent	Adsorbate	pH	Dosage (g L^−1^)	T (K)	q_max_ (mg g^−1^)	S_BET_ (m^2^ g^−1^)	Reference
activated carbon–chitosan beads	diclofenac	6	1.5	313	99.29		[1]
alginate–carbon films	diclofenac	3	1	303	37.01	35.16	[33]
alginate (Alg) composite beads with activated carbon (AC)	ibuprofen				39.6		[34]
alginate (Alg) composite beads with activated carbon (AC) and carboxymethyl cellulose (CMC)	ibuprofen				48.1		[34]
calcium chloride-caged acid-activated tamarind seed and bentonite alginate beads	ibuprofen				17.54		[35]
alginate/polypyrrole/ZnFe_2_O_4_ beads	ibuprofen	7		298	108.2		[36]
alginate/polypyrrole/ZnFe_2_O_4_ beads	acetaminophen	7		298	106.7		[36]
zirconium allied alginate beads	ibuprofen				23.33		[37]
alginate hydrogel beads of magnetic graphene oxide@MIL-88 metal–organic framework	naproxen			298	55.55		[38]
polyethyleneimine-modified magnetic sugarcane bagasse cellulose film	ibuprofen	4	0.25	318	370.52		[39]
guava seed-activated carbon-loaded calcium alginate aerogel	diclofenac				489.97	738.82	[40]

## Data Availability

The data and samples are available from the authors.

## Supplementary Materials

The following supporting information can be downloaded at https://www.mdpi.com/article/10.3390/ma18030629/s1, Figure S1: Deconvoluted C 1s and O 1s high-resolution core-level XPS spectra (AlgNa); Figure S2: Deconvoluted C 1s, O 1s, and Ca 2p high-resolution core-level XPS spectra (AlgCa); Figure S3: Deconvoluted C 1s and O 1s high-resolution core-level XPS spectra (Cel); Figure S4: Deconvoluted C 1s, O 1s, and Ca 2p high-resolution core-level XPS spectra (AlgCa + Cel); Figure S5: Deconvoluted C 1s and O 1s high-resolution core-level XPS spectra (C); Figure S6: Deconvoluted C 1s, O 1s, and Ca 2p high-resolution core-level XPS spectra (AlgCa + C); Table S1: The thermal decomposition data of AlgNa, AlgCa, Cel, C, AlgCa + Cel, and AlgCa + C.
