# Supplementary material for: Preparation and Study of Physicochemical and Adsorption Properties of Alginate Composites
Source: Materials (Basel). 2025 Jan 30;18(3):629. doi: 10.3390/ma18030629 (PMC11818849; doi:10.3390/ma18030629)
Supplement: Supplementary file 1 [file materials-18-00629-s001.zip › materials-3373553-supplementary.pdf]

Supplementary material for the paper:

Preparation and study of physicochemical and adsorption properties of alginate composites

Małgorzata Wasilewska [1,*], Sylwia Gubernat [2] and Paulina Gil-Kulik [3]

[1] Department of Physical Chemistry, Institute of Chemical Sciences, Maria Curie-Sklodowska University, Maria Curie-Sklodowska Sq. 3, 20-031 Lublin, Poland
[2] Inżynieria Rzeszów S.A., ul. Podkarpacka 59A, 35-082 Rzeszów, Poland; sylwiagubernat44@gmail.com
[3] Department of Clinical Genetics, Medical University of Lublin, 11 Radziwillowska Str., 20-080 Lublin, Poland; pgil.poczt@vp.pl
* Correspondence: malgorzata.wasilewska@mail.umcs.pl; Tel.: +48-815-375-646

3.3. XPS

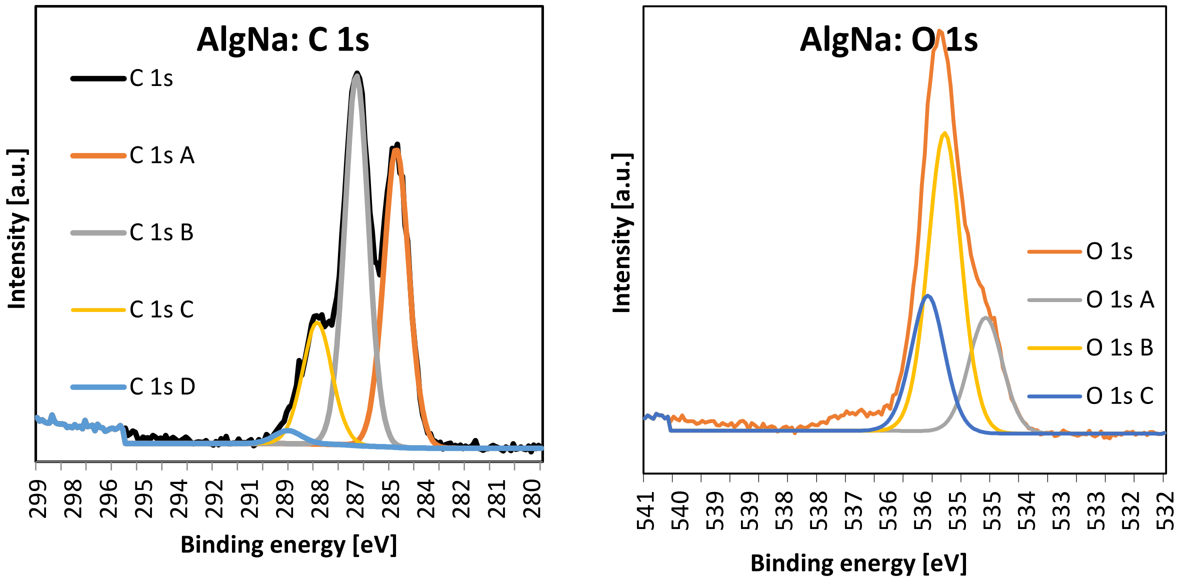

(**a**) (**b**)

Figure S1. Deconvoluted C 1s and O 1s high resolution core-level XPS spectra AlgNa.

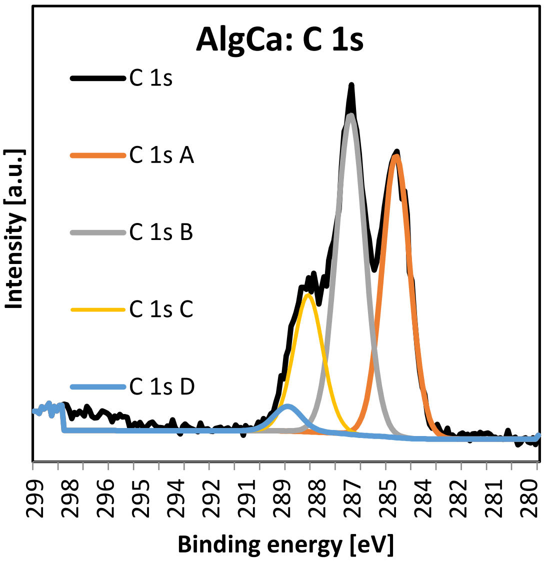

(**a**)

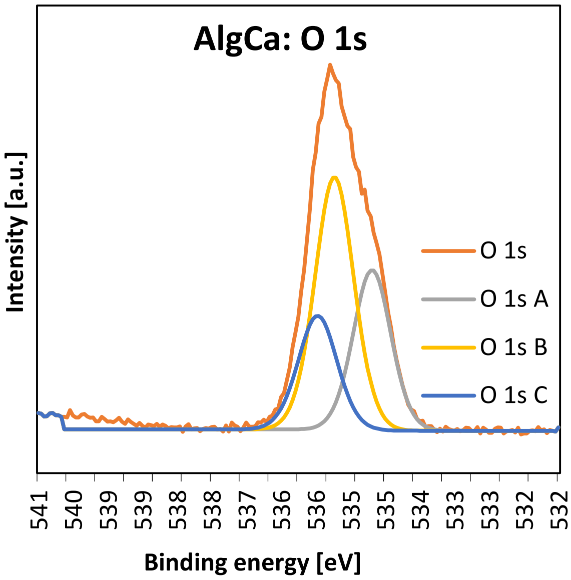

(**b**)

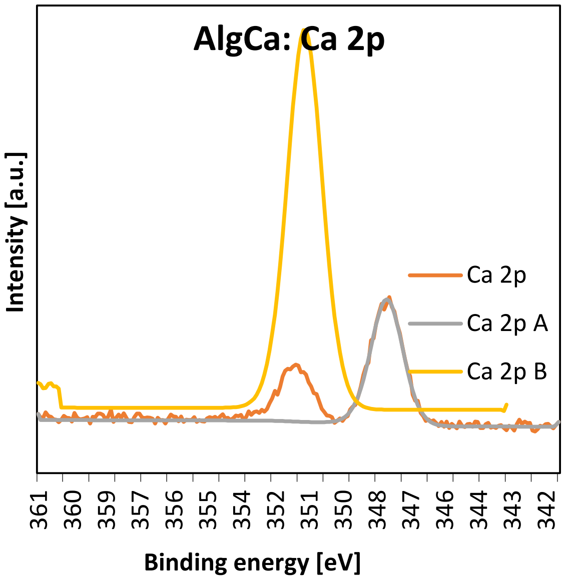

(**c**)

Figure S2. Deconvoluted C 1s, O 1s and Ca 2p high resolution core-level XPS spectra AlgCa.

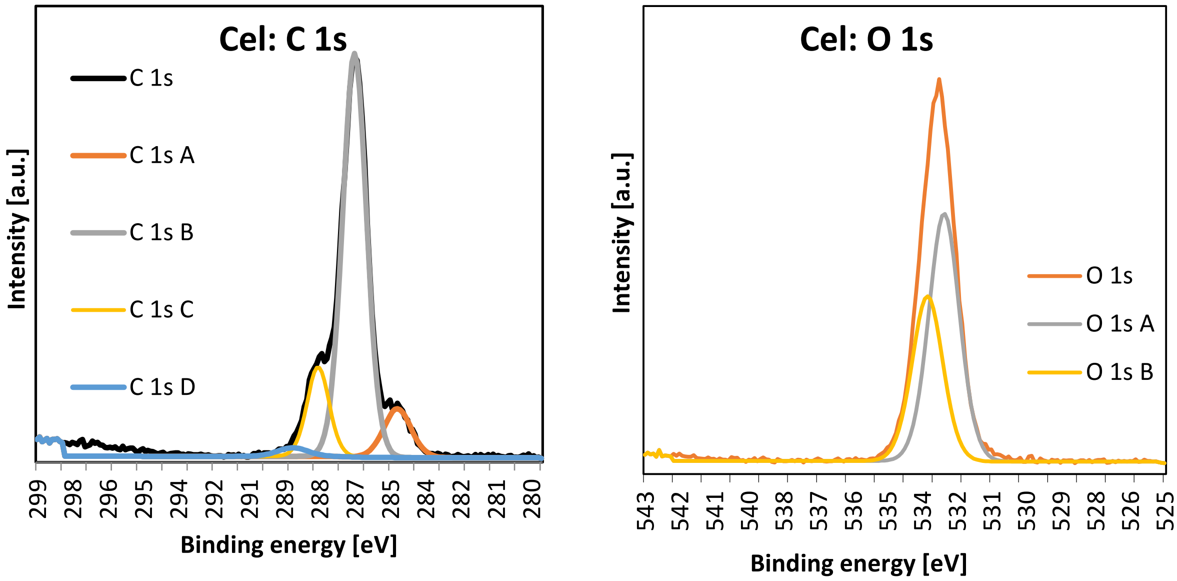

(a) (b)

Figure S3. Deconvoluted C 1s and O 1s high resolution core-level XPS spectra Cel.

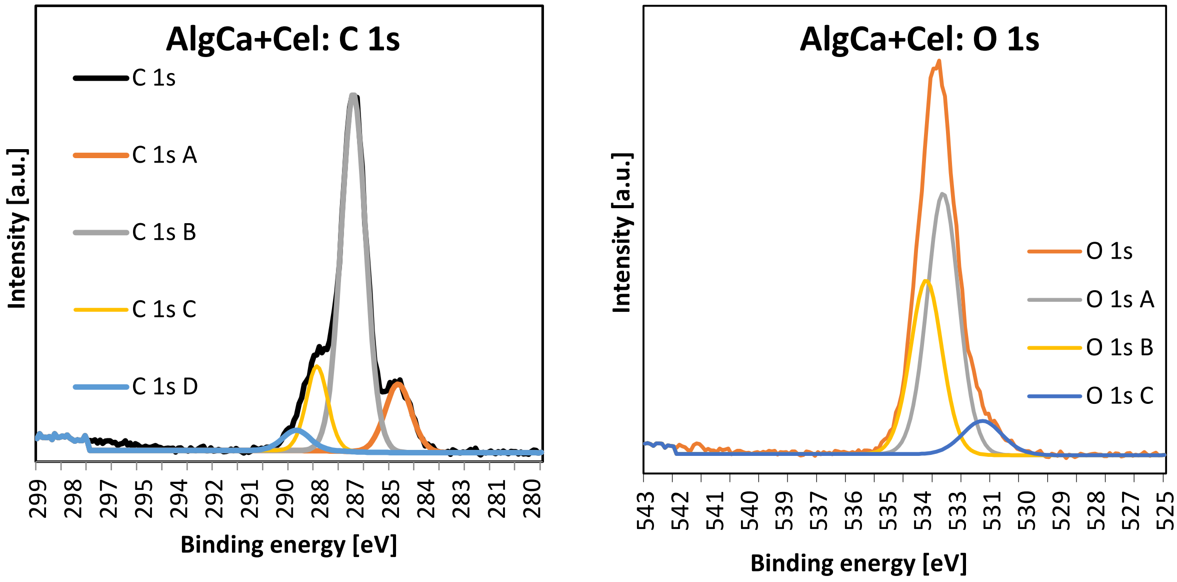

(a) (b)

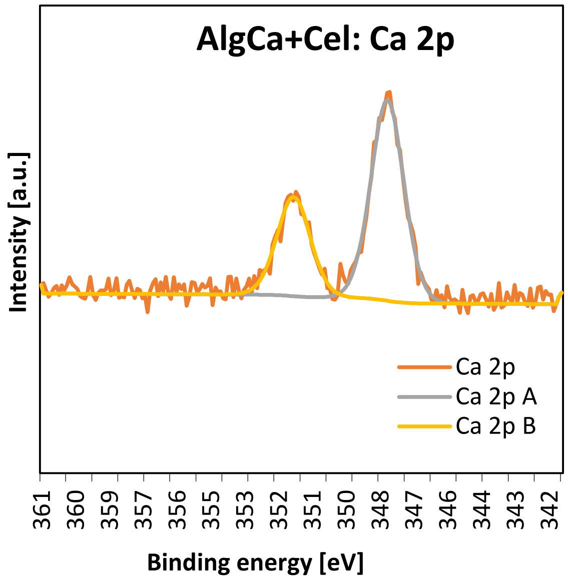

(**c**)

Figure S4. Deconvoluted C 1s, O 1s and Ca 2p high resolution core-level XPS spectra AlgCa+Cel.

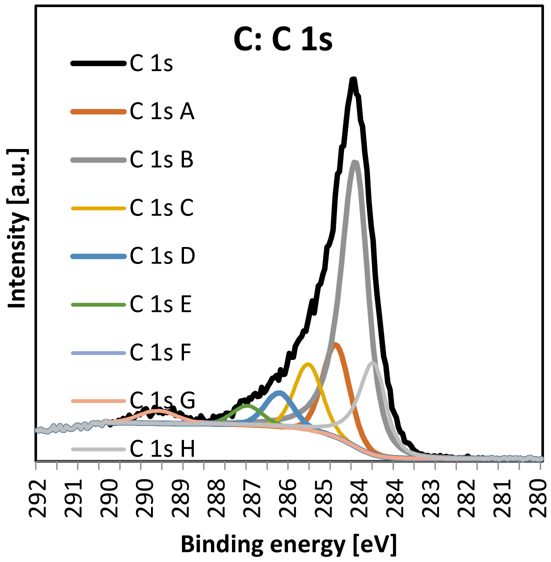

(**a**)

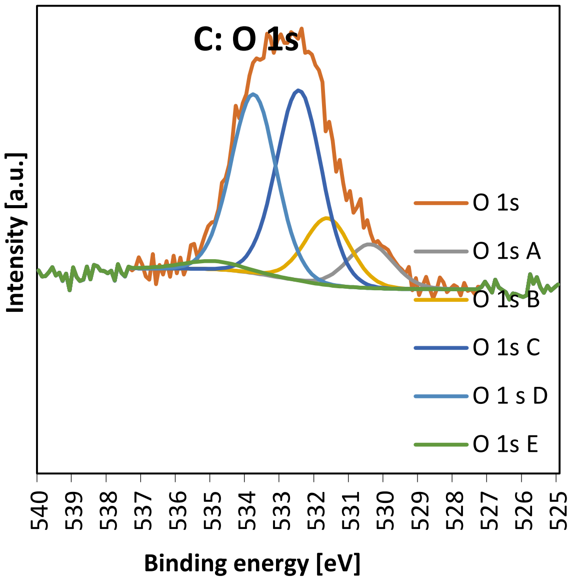

(**b**)

Figure S5. Deconvoluted C 1s and O 1s high resolution core-level XPS spectra C.

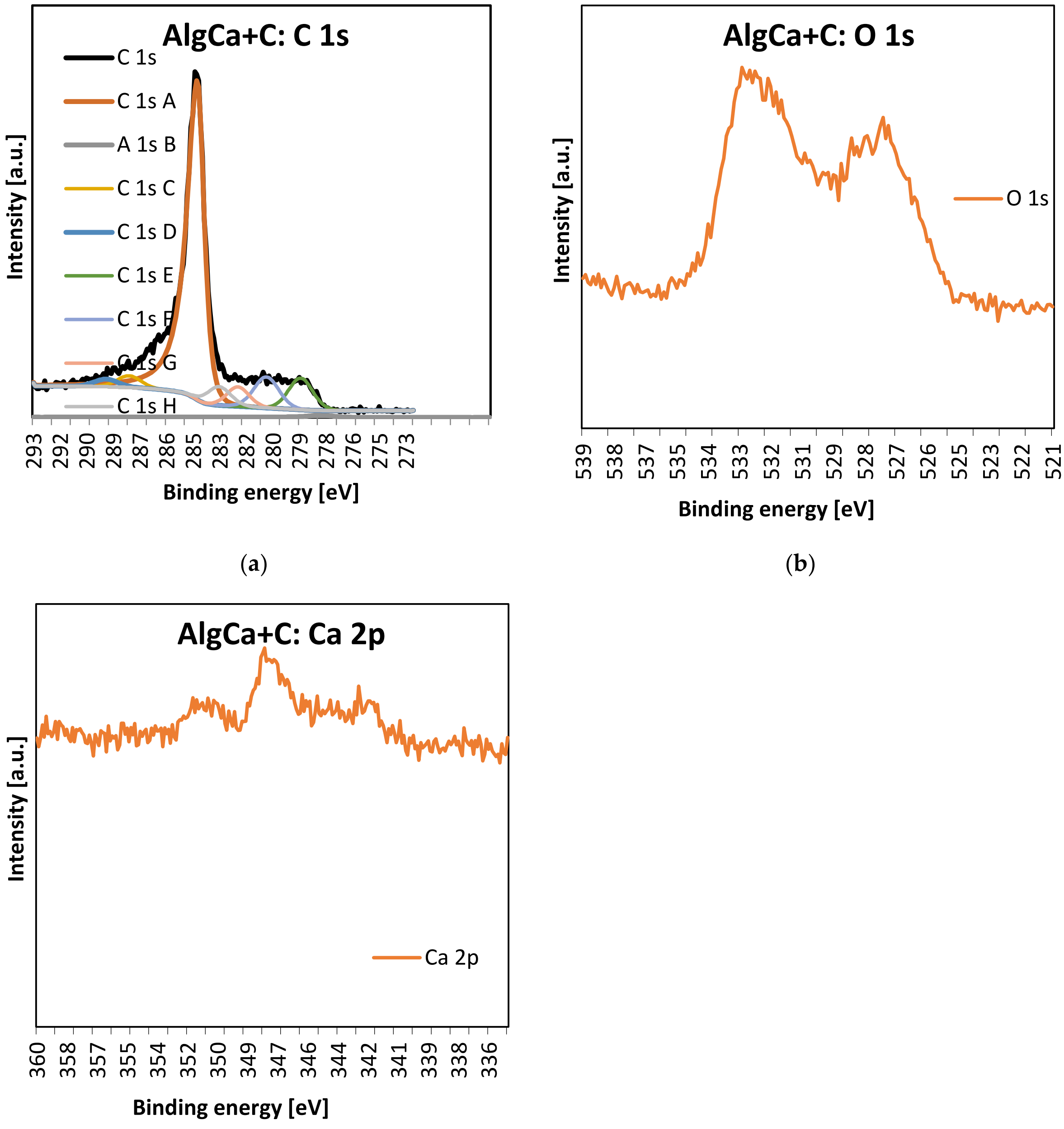

(**a**) (**b**) (**c**)

Figure S6. Deconvoluted C 1s, O 1s and Ca 2p high resolution core-level XPS spectra AlgCa+C.

3.4. Thermal analysis

Table S1. The thermal decomposition data of AlgNa, AlgCa, Cel, C, AlgCa+Cel and AlgCa+C.

Sample	TG			DTG	DSC
	ΔT [°C]	Mass Loss [%]	Total Mass Loss [%]	T_{min} [°C]	endo/exo
AlgCa	30–200	11.23	79.45	53.3	endo
	200–950	68.22		200.3	exo
					exo

				295.3	exo
				540.3	exo
				710.3	
C	30–200	1.82	91.48	94.34	endo
	200–950	89.66		654.34	exo
				714.34	exo
Cel	30–200	0.95	97.7	96.48	endo
	200–950	96.75		336.48	endo
				501.48	exo
AlgCa+C	30–200	4.31	91.47	89.52	endo
				259.52	exo
	200–950	87.16		414.52	exo
				449.52	exo
AlgCa+Cel	30–200	3.79	94.51	99.79	endo
				294.79	exo
	200–950	90.72		444.79	exo
				669.79	exo
